# Phase separation in neurodegenerative disorders: a metabolic perspective on protein aggregation and therapeutic targeting

**DOI:** 10.1186/s40035-026-00575-z

**Published:** 2026-08-31

**Authors:** Shujun Peng, Hui Chen, Guowei Yin, Alexei Verkhratsky, Chenju Yi

**Affiliations:** 1 Department of Geriatrics, Seventh Affiliated Hospital of Sun Yat-Sen University, Shenzhen, 518107 China; 2 School of Life Sciences, Faculty of Science, University of Technology Sydney, Sydney, Australia; 3 Faculty of Biology, Medicine and Health, The University of Manchester, Manchester, UK; 4 Department of Forensic Analytical Toxicology, School of Forensic Medicine, China Medical University, Shenyang, 110122 China; 5 Guangdong Provincial Key Laboratory of Digestive Cancer Research, Shenzhen, 518107 China; 6 Tianfu Jincheng Laboratory, Chengdu, 610212 China; 7 Shenzhen Key Laboratory of Chinese Medicine Active Substance Screening and Translational Research, Shenzhen, 518107 China

**Keywords:** Phase separation, Metabolic enzymes, Neurodegenerative diseases, Pathological protein aggregation, Cognition

## Abstract

Biomolecular condensates formed via liquid–liquid phase separation (LLPS) are increasingly recognised as dynamic organisers of intracellular biochemistry, particularly in neurons where spatially restricted signalling, RNA metabolism, and proteostasis are essential. Aberrant phase transitions of disease-associated proteins, including TDP-43, FUS, tau, and α-synuclein, contribute to protein aggregation and neurodegenerative pathology. Beyond protein-intrinsic sequence features, metabolic state has emerged as an important contextual regulator of condensate assembly, material properties, and liquid-to-solid maturation. Metabolic cues, including ATP availability, NAD^+^/NADH balance, redox state, lipid composition, enzyme-mediated post-translational modifications, and cellular stress responses, can influence the phase behaviour across biochemical, cellular, and disease-model systems. However, direct causal evidence in neurons, animal models, and human neurodegenerative diseases remains uneven and protein-specific. Here, we review LLPS in neurodegenerative disorders from the metabolic perspective, distinguishing established mechanisms from plausible but incompletely validated links. We discuss how pathological condensates may impair RNA metabolism, synaptic function, proteostasis, and cognition, and critically evaluate emerging therapeutic strategies that aim to modulate aberrant phase behaviour. This review therefore provides a cautious framework in which metabolic dysregulation is considered a potential upstream contributor to pathological phase transitions rather than an established master regulator.

## Introduction

Neurodegenerative diseases are a diverse group of disorders characterised by the progressive loss of neurons, translating into damage to brain structure and loss of function, posing a major challenge to global health. Alzheimer’s disease (AD), Parkinson’s disease (PD), amyotrophic lateral sclerosis (ALS), and frontotemporal lobar degeneration (FTLD) involve the accumulation of misfolded proteins, leading to neuronal death and, ultimately, cognitive decline and motor impairment [1–3]. Despite intense investigation, the molecular mechanisms governing protein misfolding and aggregation remain incompletely understood. Recently, liquid–liquid phase separation (LLPS) emerged as a novel concept in understanding cell structure and its pathological modifications. In biophysical terms, LLPS is a reversible demixing process that concentrates selected proteins and nucleic acids into condensed phases separated from cytoplasm or nucleoplasm and not enclosed by lipid membranes [4–6]. These condensates contribute to and regulate different biological processes such as gene expression, RNA metabolism, signal transduction, and stress responses [7].

In the central nervous system (CNS), the dynamic regulation of LLPS is essential for neuronal homeostasis. Synaptic plasticity, which underlies learning and memory, depends on the precise spatiotemporal organisation of proteins and RNA within phase-separated compartments. These condensates organise receptors, scaffold proteins, and signalling enzymes into functional nanodomains, while RNA granules support localised translation [8, 9]. At presynaptic terminals, vesicle-associated protein synapsin-1 forms condensates and recruits lipid vesicles into these condensates to regulate vesicle clustering [10]. In parallel, RNA metabolism and proteostasis are coordinated within stress granules and processing bodies, further highlighting the role of LLPS in maintaining neuronal homeostasis [11, 12]. Under pathological conditions, dysregulated phase separation contributes to the formation of aberrant protein aggregates that drive neurodegeneration. Many disease-associated proteins, including tau in AD, α-synuclein in PD and dementia with Lewy bodies, as well as Fused in sarcoma (FUS) and TAR DNA-binding protein 43 (TDP-43) in ALS and FTLD, contain intrinsically disordered regions (IDRs) that mediate weak multivalent interactions and promote condensate formation. Perturbation of these processes shifts condensates from dynamic liquid-like states to solid-like aggregates, thereby contributing to disease pathogenesis [13–16]. Recent studies have suggested that intracellular ATP depletion can increase axoplasmic viscosity and accelerate the pathological condensation of proteins associated with the pathogenesis of PD and ALS. These findings suggest a potential functional link between energy metabolism and protein aggregation in neurons [17].

While the role of LLPS in neurodegeneration is increasingly established, critical knowledge gaps remain. Most studies have focused on intrinsic properties such as sequence complexity and disordered conformation that drive phase separation. However, the role of environmental factors, including cellular metabolism, in the regulation of pathological phase separation remains undercharacterised. Evidence for the metabolism–LLPS coupling currently spans several levels: (i) biochemical and reconstituted systems have demonstrated direct metabolite–protein interactions, such as ATP-mediated regulation of FUS, TDP-43, and α-synuclein phase behaviour; (ii) cellular stress models have shown altered condensate dynamics under ATP depletion, oxidative stress, endoplasmic reticulum (ER) stress, or altered post-translational modification (PTM) signaling; (iii) animal models have linked metabolic perturbation or glycogen/lipid imbalance to neurodegenerative phenotypes; and (iv) other disease models have demonstrated the co-existence of metabolic abnormalities and pathological condensates or aggregates. Accordingly, in this review we propose metabolic dysregulation as an emerging and context-dependent upstream influence on condensate behaviour.

**Fig. 1:**
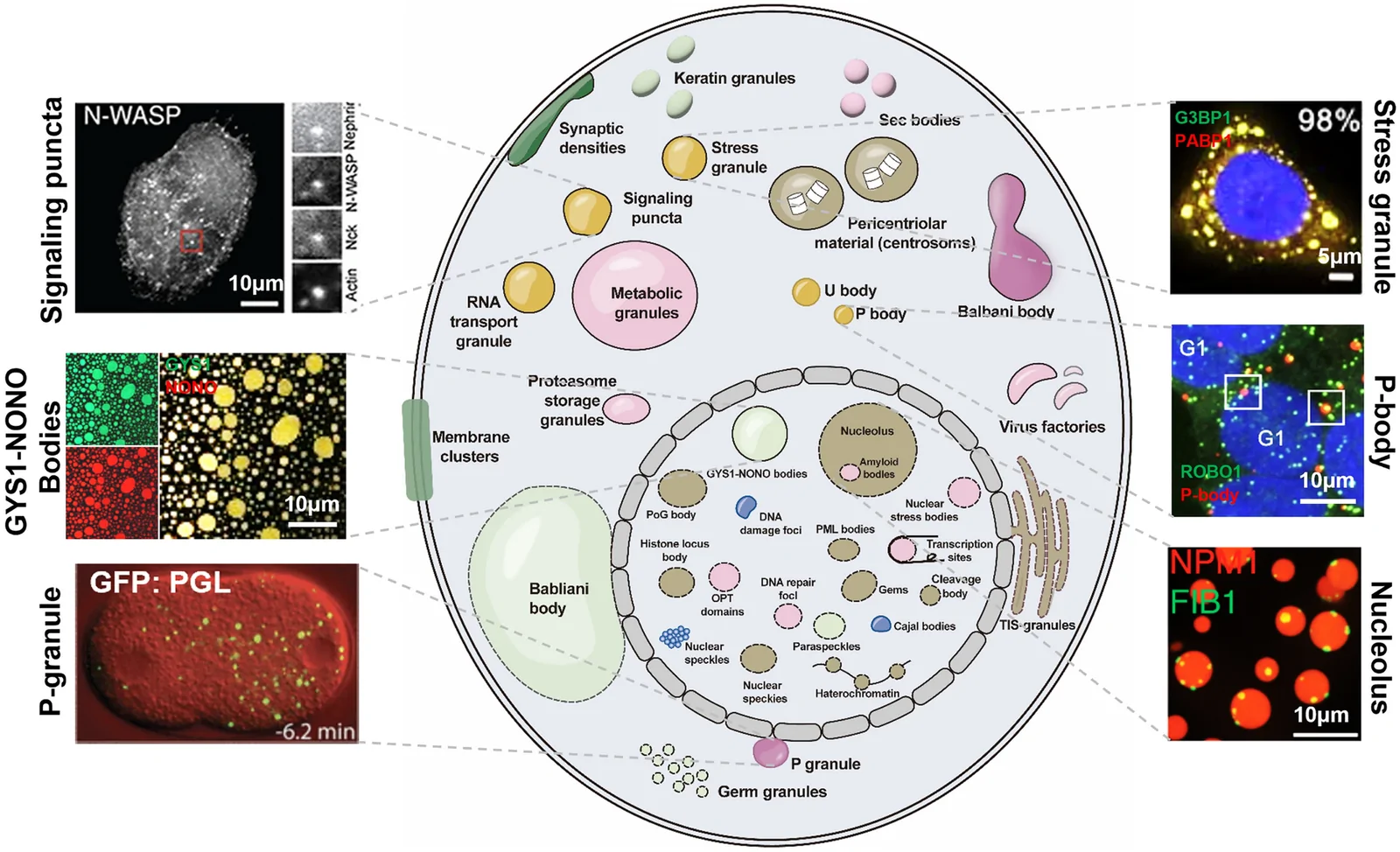
LLPS in cell biology. The schematic illustrates several membrane-less organelles in an eukaryotic cell at different locations (nucleus, cytoplasm, and membranes) that are formed through the process of LLPS. The inset shows the experimental observation of membrane-less organelles, including signaling puncta, GYS1-NONO bodies, P granules, stress granule, P body, and nucleolus [18–22]. The signaling puncta image was reproduced from [19] with permission from The American Association for the Advancement of Science, Copyright © 2019. The GYS1-NONO body image was reproduced from [18] with permission from Springer Nature, on behalf of CDDpress, Copyright © 2025. The P-granules image was reproduced from [20] with permission from The American Association for the Advancement of Science, Copyright © 2009. The stress granule image was reproduced from [21] under the terms of the Creative Commons Attribution License. Copyright © 2016. The P body image was reproduced from [22] with permission from Elsevier Inc. Copyright © 2024. The nucleolus image was reproduced from [23] with permission from Elsevier Inc. Copyright © 2016

This review aims to summarise recent advances connecting dysregulated condensates with cognitive decline, while highlighting emerging approaches to mitigating neurodegenerative diseases. We first outline the molecular basis of physiological and pathological LLPS. We then evaluate metabolic regulations on LLPS and disease‑related protein aggregation, including ATP and nucleotide metabolism, glycogen-related pathways, ER stress, PTM enzymes, mitochondrial redox balance, glucose hypometabolism, and lipid-membrane biology. We further recapitulate crosstalks between LLPS and multiple pathophysiological processes, integrating evidence from biochemical, biophysical, cellular, animal-model, and human disease studies. Finally, we discuss potential therapeutic strategies targeting LLPS-mediated mechanisms and emphasise translational challenges, such as CNS delivery, blood–brain barrier (BBB) penetration, dosing, specificity, and long-term safety. To provide a conceptual and spatial overview for the following sections, Fig. 1 summarises representative biomolecular condensates across subcellular compartments and illustrates how LLPS underpins diverse physiological processes.

## Molecular mechanisms of LLPS in health and disease

LLPS represents a fundamental principle of cellular organisation, by which dynamic and membraneless compartments are formed within cells to facilitate biochemical reactions, cellular processes, and in particular coordinate local translation, synaptic vesicle organisation, and stress responses in neurons [4–6]. Under physiological conditions, these biomolecular condensates, such as nucleoli, stress granules, and P-bodies, maintain cellular homeostasis, and regulate gene expression, RNA metabolism, and signal transmission [24, 25]. The formation of these cellular condensates is driven by multivalent interactions such as hydrophobic interactions, electrostatic interactions, π-π stacking and cation-π interactions between IDRs of proteins or between proteins and nucleic acids [26–30]. RNA abundance, ATP, salt concentration, pH, molecular crowding, and PTMs further tune the nucleation, growth, fusion, and dissolution. One example is the ‘stickers-and-spacers’ model, which explains how certain interaction motifs known as stickers are separated by flexible spacers within IDRs, enabling multiple interactions that result in condensate formation [31, 32].

The functional versatility of LLPS is particularly pronounced in the intricate architecture of neurons. For instance, RNA granules act as dynamic membraneless organelles that orchestrate the spatiotemporal regulation of mRNA transport and local translation within dendritic compartments, thereby critically modulating synaptic plasticity during neuronal activity [9, 11]. Similarly, at presynaptic nerve terminals, LLPS underpins the assembly of release-site scaffolds and synaptic vesicle clusters. Synapsin-1 forms condensates that capture presynaptic vesicles, while phosphorylation by CaMKII disperses and suppresses the condensates in a manner resembling activity-dependent vesicle mobilisation [10]. More broadly, presynaptic condensates help coordinate synaptic vesicle availability with calcium-channel coupling and vesicle fusion [33]. The dynamic properties of these condensates, demonstrated by rapid molecular turnover and liquid-like fluidity, are critical determinants governing their physiological functions [34]. However, disruption of this balance may cause abnormal LLPS and pathological protein aggregates, linked to neurodegeneration [3, 14, 15].

### Pathological phase separation of disease-associated proteins

**Fig. 2:**
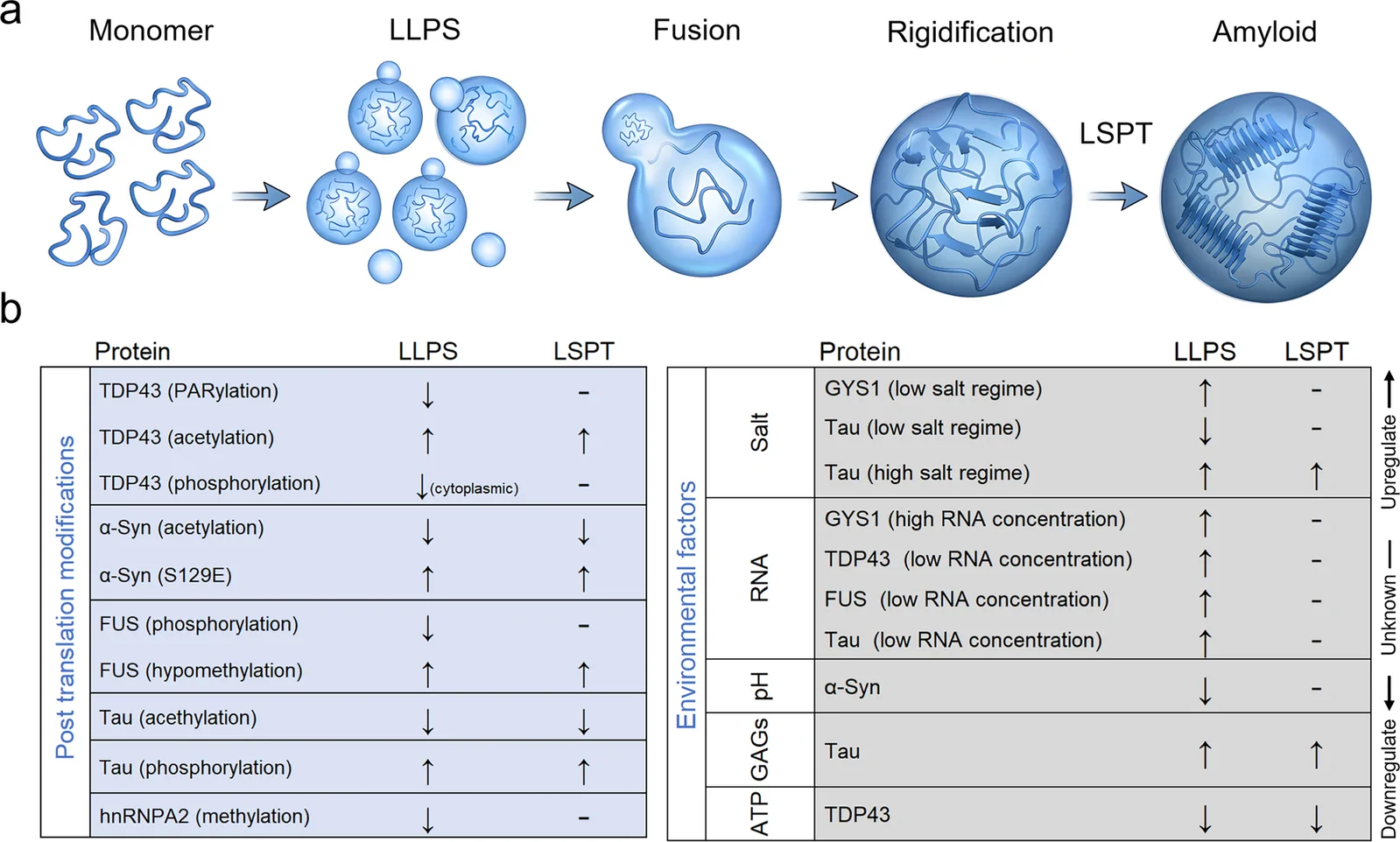
Schematic representation of different effectors modulating LLPS and liquid-to-solid phase transition (LSPT) by various proteins. **a** Schematic representation showing LLPS and LSPT of proteins. **b** A table summarising the effects of PTMs and environmental factors on LLPS and LSPT for major neurodegeneration-related proteins. Upward arrow indicates increased LLPS propensity, while the downward arrow indicates reduced LLPS propensity, and the dash indicates no information available yet

Aberrant LLPS is increasingly recognised as a critical step in the pathogenesis of AD, PD and dementia with Lewy bodies, often leading to the irreversible aggregation of toxic amyloid fibrils or amorphous inclusions [35–37]. The transition from liquid-like condensates to solid-like aggregates, known as ‘maturation’ or ‘ageing’ of condensates, is a central pathological process due to restricted mobility and increased frequency of intermolecular interactions, along with more extended conformations in condensates [3, 38]. This section examines the molecular mechanisms of LLPS in disease-related protein aggregation, emphasising the transition from physiological condensates to pathological states. As summarised in Fig. 2, diverse regulatory inputs, including acetylation, phosphorylation, RNA concentration, ionic strength, and ATP levels, differentially modulate LLPS propensity and liquid-to-solid phase transition across major neurodegeneration-associated proteins [7, 8, 39].

#### TDP-43

Cytoplasmic aggregation of RNA-binding protein TDP-43 is a pathological hallmark in nearly all cases of ALS and about half of FTLD cases [13, 16, 40]. In these disorders, the intrinsically disordered C-terminal low-complexity domain (LCD) of TDP-43 exhibits a strong propensity for LLPS and subsequent aggregation [35, 41]. The LCD can form dynamic, reversible condensates that progressively mature into fibrillar aggregates, suggesting that weak multivalent interactions lower the energetic barrier for droplet formation while simultaneously predisposing condensates to pathological solidification [35]. Recent advances in cryo-electron microscopy (cryo-EM) have provided detailed insights into the structural architecture of TDP-43 aggregates, revealing a tightly packed amyloid core characterised by a planar C-terminal region and a non-planar N-terminal region, which distinguishes full-length assemblies from shorter fragments [42]. Notably, TDP-43 fibrils exhibit pronounced structural heterogeneity, manifested as multiple distinct conformations that differ in protofilament number, orientation, and packing arrangements [43, 44]. This heterogeneity is further shaped by PTMs, particularly phosphorylation, as well as ALS/FTLD-associated mutations, which collectively influence assembly propensity, protofilament organisation, and fibril maturation [42, 43, 45, 46]. Importantly, these structurally distinct fibril polymorphs likely confer differential seeding capacities, stability, and interaction interfaces, thereby modulating the recruitment of cofactors and contributing to disease-specific pathological diversity.

The TDP-43 LLPS is largely governed by aromatic residues, with its α-helical segment self-assembly reducing the number of required motifs, and disease-related mutations can indirectly disrupt LLPS [41, 47, 48,]. Key hydrophobic and aromatic regions in the TDP-43 LCD that drive self-association have been identified, with specific mutations either inhibiting or promoting this process [48]. PTMs and RNA binding can also regulate TDP-43 LLPS and aggregation. Lysine acetylation impairs RNA binding and promotes aggregation [49]. Effects of phosphorylation are site- and context-dependent [46], with S48 phosphorylation disrupting TDP-43 polymerisation and LLPS [50]. Methionine-rich residues within the LCD of TDP-43 modulate its function through oxidation: H_2_O_2_ induces methionine oxidation and droplet melting, a process which can be reversed by methionine sulfoxide reductase [51]. The M337V mutation abrogates the H₂O₂-induced polymerisation, implicating its role in neurodegenerative diseases. Additionally, methionine sulfoxidation disrupts a helical motif in the prion-like domain of TDP-43, compromising phase separation, amyloid formation, and chaperone recognition [52].

Beyond these molecular regulatory mechanisms, RNA-binding deficiencies and PTMs can alter TDP-43 condensation in spinal motor neurons, affecting compartmentalisation, condensate mobility, and downstream RNA metabolism [53]. In the neuronal context, acute intracellular ATP depletion increases axoplasmic viscosity and promotes pathological protein condensation, including TDP-43-related aggregation in ALS motor neurons [17]. Nevertheless, it is unclear whether ATP loss facilitates pathological condensation or whether ATP-driven LLPS is the initiating event in human ALS. Additionally, the disrupted multimerisation of TDP-43 leads to pathological inclusions and impairs RNA processing in brains from patients with sporadic ALS [54].

#### FUS

FUS is an RNA-binding protein linked to ALS and FTLD, known for its formation into cytoplasmic inclusions [13, 16, 55]. Similar to TDP-43, the low-complexity domain of FUS (FUS-LC) contributes to LLPS and pathological aggregation [56, 57]. Recent studies have defined the mechanisms of FUS aggregation using cryo-EM, nuclear magnetic resonance (NMR) spectroscopy, single-molecule spectroscopy, pressure-jump experiments, and molecular simulations. In particular, fibrils formed by the C-terminal region of the FUS-LC (FUS-LC-C) adopt in-register parallel cross-β structures, as revealed by a 2.62 Å cryo-EM model [58]. Additionally, FUS-LC self-assembles through two cross-β cores, whose balance is disrupted by ALS-associated mutations [56]. Notably, NMR, microscopy, and simulation studies demonstrated that the tyrosine residues facilitate condensate formation by the LCD of the RNA-binding protein EWS, with distinct interaction patterns distinguishing EWS from FUS and TAF15 [59]. Single-molecule FRET revealed two conformational subpopulations of the FUS LCD, in which structural unwinding shifts intramolecular contacts to intermolecular ones [60]. A disease-related FUS P525L mutation enhances structural plasticity and interchain interactions, accelerating pathological aggregation and revealing sequence-encoded structural changes in phase separation. The dynamic mechanism has been further characterised by NMR/EPR combined with agarose hydrogels, which found FUS compaction upon droplet formation [61]. Importantly, ATP is able to suppress FUS LLPS and prevent irreversible aggregation through disrupting molecular interactions, including pathological factor-induced LLPS of ALS-linked variants R495X and P525L [62]. Together, these findings support ATP-sensitive FUS condensation as a potential metabolic vulnerability in ALS; further in vivo validation in disease-relevant models is needed.

#### Tau

Tau is a microtubule-associated protein that forms neurofibrillary tangles in AD and tauopathies [63, 64]. Tau undergoes phase separation, and its cellular condensates can further transform into pathological aggregates even before neurodegeneration occurs [63]. The phase separation of tau may be influenced by interactions with RNA, PTMs, and binding with small molecules. Native tau binds RNA with high affinity but low specificity, and the tau-RNA complexes inhibit microtubule polymerisation and drive disease-relevant changes [65]. Tau-RNA complex-related lesions are evident in various tauopathies, suggesting that tau/RNA binding may drive tau aggregation. Notably, the stress granule protein G3BP2 directly interacts with tau and suppresses its aggregation, representing an adaptive protective mechanism against tauopathies [66].

In addition to RNA-mediated regulation, PTMs also modulate tau phase separation. The GSK3β-mediated phosphorylation of tau within the proline-rich domain is essential for tau condensation, while inhibiting GSK3β activity effectively prevents aberrant tau condensation [67]. Furthermore, N-glycosylation modifies the biophysical characteristics of tau. Specifically, small glycans facilitate tau aggregation and LLPS, while larger glycans reduce these effects. High-mannose glycans at N410 enhance phosphorylation by GSK3β, suggesting a novel mechanism underlying AD pathology [68]. Additionally, the Ca^2+^-dependent chaperone activity of S100B protein influences tau LLPS, where Ca^2+^-bound S100B suppresses tau demixing and stabilises tau droplets [69]. Small molecules, such as tannic acid, can influence tau LLPS biphasically: at low tannic acid-to-tau molar ratios it promotes, while at higher ratios it inhibits LLPS [70]. This action occurs through hydrophobic interactions and hydrogen bonds. These findings suggest that targeted control of tau phase transitions could be used to develop new therapies [64].

#### α-Synuclein

α-Synuclein aggregation is a hallmark of PD and synucleinopathies, leading to Lewy body formation [37]. Its LLPS can precede amyloid development, creating hydrogels enriched in oligomers and fibrils. Low pH, phosphorylation, and familial mutations can drive α-synuclein phase separation [37], whereas multiple proteins modulate α-synuclein LLPS. For instance, β-synuclein promotes α-synuclein phase separation yet delays its fibrillisation, whereas disease-related mutations in β-synuclein abrogate this regulatory effect [71]. S100A9 interacts with α-synuclein to form mixed condensates, altering the aggregation patterns linked to neurodegenerative pathogenesis [72]. ATP may also influence α-synuclein behaviour through hydrotropic interactions. Recent NMR and molecular dynamics analyses indicate that ATP interacts with α-synuclein through both the adenine ring and the triphosphate-mediated contacts, and the predominant contribution of each ATP moiety shifts with ATP concentration. This provides a mechanism for concentration-dependent effects of ATP on α-synuclein conformation and explains how ATP regulates pathological aggregation-prone proteins under physiological conditions [73]. Lipid metabolism and membrane context are equally important. Physiologically, α-synuclein is associated with lipid membranes, and this membrane binding can increase its local concentration and alter its conformational ensemble at membrane surfaces. Under misfolding-prone conditions, these membrane-associated interactions, alongside lipid vesicles, can promote α-synuclein aggregation via protein-lipid co-aggregation mechanisms [74]. Studies on human postmortem and iPSC-derived neurons further support a link between α-synuclein abundance and dysregulation of lipid homeostasis at mitochondria-associated ER membranes [75]. In conclusion, α-synuclein phase behaviour and aggregation are shaped by a complex interplay among protein sequence, ATP availability, PTMs, lipid composition, membrane interfaces, and inflammatory co-factors.

#### Other proteins involved in neurodegeneration

The cellular prion protein (PrP^C^) is a highly conserved mammalian cell surface glycoprotein that plays a critical role in neurodegenerative diseases. PrP^C^ misfolds into the toxic scrapie isoform (PrP^Sc^) to cause infectious prion diseases and interacts with aggregation-prone proteins, such as amyloid-β, to drive AD pathology [76, 77]. PrP^C^ undergoes a conformational transition following LLPS, which may represent an early molecular event facilitating the formation of pathogenic PrP conformers in prion diseases and associated neurodegenerative disorders [78]. Proteolysis promotes the aggregation of C2-PrP, a N-terminally truncated PrP fragment, only when the full-length PrP has formed biomolecular condensates via LLPS before proteolysis. In addition, the chaperone Clusterin can prevent the aggregation of C2-PrP after proteolysis and interfere with aberrant PrP phase transition in vitro [79]. Beyond the PrP-related regulatory mechanisms, WDR45, which plays a critical role in the rare X-linked neurodegenerative disorder BPAN (β-propeller protein-associated neurodegeneration), has been identified as a critical regulator of stress granule disassembly. Specifically, WDR45 forms gel-like condensates through its WD5 domain, competitively displacing G3BP1 from Caprin-1 to facilitate stress granule clearance. Notably, BPAN-associated mutations in WDR45 compromise its capacity to form condensates and interact with Caprin-1, leading to impaired stress granule disassembly [80]. Additionally, RBM33 LLPS is linked to aging and microglial senescence through its binding to the *CDKN1A* promoter [81]. Furthermore, the ER chaperone GRP78 undergoes LLPS under ER stress and recruits SOD1(A4V), but current evidence supports a bidirectional, context-dependent role in programming aggregation dynamics rather than a simple protective or toxic mechanism [82]. Collectively, these findings indicate that phase separation is broadly linked to pathological aggregates, although the mechanistic depth and causal evidence differ considerably across proteins.

## Metabolic regulation of LLPS and protein aggregation

**Fig. 3:**
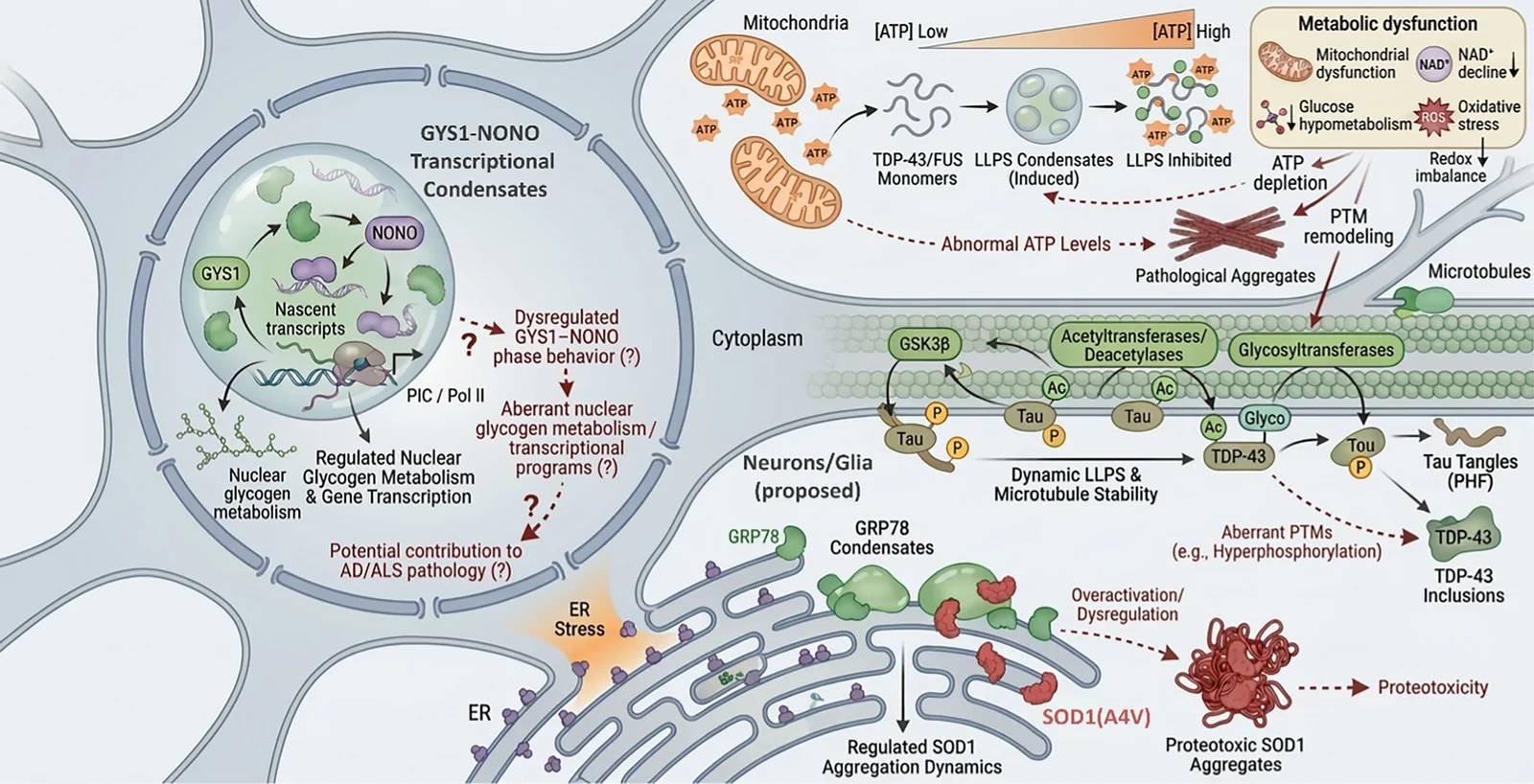
Metabolic regulations of LLPS in neurodegeneration. The schematic illustrates how metabolic dysfunction may reshape the LLPS dynamics and pathological protein aggregation through interconnected bioenergetic, redox, and post-translational mechanisms. Mitochondrial dysfunction, NAD⁺ decline, glucose hypometabolism, and oxidative stress may converge on ATP depletion, redox imbalance, and PTM remodelling. ATP acts as a hydrotrope and energy-related regulator of condensate material properties, with reduced ATP availability promoting TDP-43/FUS condensation and higher ATP levels suppressing aberrant LLPS. The redox imbalance may further influence redox-sensitive residues and the aggregation propensity. In the cytoplasm, metabolically linked PTM enzymes, including GSK3β, acetyltransferases/deacetylases, and glycosyltransferases, regulate the phase behaviour, microtubule association, and pathological aggregation of tau and TDP-43. Under ER stress, GRP78 forms condensates that recruit SOD1(A4V) and modulate its aggregation dynamics; however, overactivation or dysregulation of this pathway may contribute to proteotoxic SOD1 aggregation. In the nucleus, GYS1–NONO transcriptional condensates regulate nuclear glycogen metabolism and gene transcription, whereas their potential relevance to neuronal/glial vulnerability and AD/ALS pathology remains hypothetical. Dashed arrows and question marks indicate proposed disease-associated mechanisms requiring further experimental validation

Neuronal health depends on the interplay between cellular metabolism and protein homeostasis. When metabolic homeostasis is interrupted, changes in ATP concentration, redox balance, pH, ionic strength, metabolite availability, lipid composition, enzyme activity, and stress signalling can lead to alterations of protein folding, PTMs, intermolecular interactions, and properties of condensate materials. In this section, we cover classical metabolic concepts including ATP, PTMs, ER stress, glycogen-associated mechanisms, mitochondrial metabolism, NAD^+^/NADH balance, glucose hypometabolism, lipid-membrane biology, and redox-sensitive control of protein phase behaviour. We emphasise that the strength of evidence differs by mechanism. Direct metabolite–protein effects, particularly those involving ATP and selected proteins, are best supported in reconstituted systems, whereas cellular and neuronal evidence is still emerging. Animal and human evidence often link metabolic dysregulation with protein aggregation, but do not consistently establish a direct causal connection through LLPS. Thus, these disease-associated phase transitions should be viewed within a broader metabolic context that can influence condensate assembly, material properties, and maturation, as outlined in Fig. 3.

### ATP as a regulator of LLPS

ATP is both a key indicator of cellular energy status and a physicochemical regulator of biomolecular phase behaviour. In addition to its canonical role as an energy substance, ATP functions as a biological hydrotrope that regulates protein solubility and can suppress aberrant phase transitions at millimolar concentrations [83]. Proteome-wide solubility and thermal stability profiling further showed that ATP affects protein structure, protein-complex stability, and the solubility of many positively charged, pathological nucleic-acid-binding proteins that are associated with membraneless organelles [84]. For TDP-43, ATP binds the arginine residues in its prion-like domain and produces concentration-dependent, biphasic modulation of LLPS: lower ATP concentrations can promote condensate formation and higher concentrations inhibit phase separation [85]. For FUS, ATP competes with nucleic acids and modulates condensate formation, suppresses fibrillisation of the RNA-recognition motif of FUS, and inhibits FUS LLPS and irreversible aggregation of ALS-linked variants through synergistic contributions from its adenosine and triphosphate moieties [62, 86]. For α-synuclein, ATP has multisite and moiety-specific interactions with α-synuclein. The triphosphate-mediated contacts at N-terminal lysine residues of α-synuclein disrupt long-range intramolecular contacts, resulting in conformational expansion of α-synuclein. This provides a molecular basis for hydrotropic modulation of an aggregation-prone intrinsically disordered protein (IDP) [73]. Importantly, in neurons, evidence now suggests that intracellular ATP depletion can increase the axoplasmic viscosity and promote pathological protein condensation in mammalian neurons, including disease-relevant models of ALS and PD [17]. These findings support ATP as a metabolite linking bioenergetics to the properties of condensate material. However, whether chronic ATP insufficiency can initiate, exacerbate, or merely co-exist with pathological LLPS in human neurodegenerative disease remains unresolved.

### Glycogen synthase 1 (GYS1) LLPS and glycogen metabolism

Glycogen metabolic homeostasis is vital for cellular energy storage and function, especially in energy-demanding tissues, such as muscles and neurons. We previously identified GYS1, a key glycogenic enzyme, as a phase-separating protein that forms nuclear condensates with NONO/p54nrb and MyoD to regulate nuclear glycogen synthesis, myogenic differentiation, and muscle regeneration [18]. This finding provides a proof-of-principle that metabolic enzymes can form nuclear condensates and coordinate local metabolism with transcriptional programs. At present, its relevance to neurodegeneration is hypothetical rather than established, because direct evidence for the presence of GYS1-NONO condensates in neurons, glia, brain tissue, or neurodegenerative disease models remains limited. A broader link between glycogen metabolism and neurodegeneration is supported by animal studies showing that pathological glycogen accumulation can accelerate or modify disease phenotypes in animal models of ALS, Lafora disease, and AD [87–89]. Furthermore, NONO is a nuclear protein with core functions in pre-mRNA splicing, transcriptional regulation, and RNA stability [90]. Nuclear pathological inclusions comprising NONO/SFPQ and adenosine-to-inosine-edited mRNAs have been implicated in PD and dementia with Lewy bodies [91]. Thus, the possible GYS1-NONO axis may provide a conceptual bridge between glycogen metabolism, nuclear RNA processing, and condensate biology. This hypothesis remains to be tested directly in nervous systems.

### ER stress and GRP78 LLPS

The ER functions as a central hub for protein folding, modification, and trafficking. ER stress, typically triggered by the accumulation of misfolded proteins, is a common hallmark of neurodegenerative diseases. Emerging evidence suggests a direct mechanistic link between ER stress and LLPS, highlighting a previously unrecognised role of protein quality control. Recent studies demonstrated that the ER-resident chaperone GRP78 undergoes phase separation under ER stress conditions and can recruit ALS-associated protein SOD1(A4V) [82]. The consequence of this recruitment is best described as context-dependent. During an acute or controlled stress response in the ER, GRP78 condensates may help compartmentalize misfolded proteins, increase the local chaperone concentration, and facilitate isolation or degradation. Under prolonged stress, however, the same condensates may create a locally concentrated microenvironment that favours aberrant maturation, altered aggregate polarity, or persistent misfolded protein assemblies, leading to ER stress and neural toxicity. Current evidence suggests that GRP78 LLPS may have a directional, context-dependent influence on SOD1(A4V) aggregation, as it reshapes the local physicochemical environment of misfolded proteins without conferring a uniformly protective or toxic role. In addition, as an ATP-dependent chaperone, GRP78 couples the ATPase-driven substrate binding and release cycle to protein folding. Notably, GRP78 condensates recruit the ALS-associated mutant protein SOD1(A4V), thereby modulating its aggregation dynamics [82]. This process is accompanied by local changes in the physicochemical environment, including polarity shifts within SOD1(A4V) aggregates, suggesting that GRP78 condensates actively reshape the microenvironment of misfolded proteins to influence their assembly behaviour. Mechanistically, GRP78-mediated phase separation appears to facilitate the spatial organisation of misfolded proteins, promoting their sequestration and processing within condensates. In addition, GRP78 may retain ATP-dependent chaperone activity within condensates, where ATP hydrolysis-driven conformational cycles regulate substrate binding and release. This activity may allow GRP78 condensates to serve as a dynamic platform linking ATP-dependent energy utilisation, ER stress responses, and proteostasis maintenance [92]. Validations in neurons, glia, and ALS animal models are required to determine their overall impact in disease [82]. Collectively, these findings indicate that ER stress-induced LLPS of GRP78 serves as an underlying mechanism for the compartmentalisation and regulation of misfolded proteins. This highlights a broader principle whereby metabolic enzymes and chaperones exploit phase separation to couple cellular stress responses with proteostasis, thereby linking ER stress, metabolism, and condensate dynamics in neurodegenerative disease pathogenesis.

### PTMs as enzyme-driven modulators

PTMs, which are covalent protein modifications by specific enzymes, play a key role in controlling protein function, localisation, and interactions. Most PTMs are metabolically coupled because they depend on ATP, acetyl-CoA, S-adenosylmethionine, NAD^+^/NADH, UDP-sugars, redox-active cofactors, or kinase/phosphatase signalling. PTMs of pathological proteins, especially phosphorylated forms, are common in neurodegenerative diseases and can reshape the phase behaviour. For example, hyperphosphorylation of tau mediated by GSK3β is important for tau condensation during tauopathies [67]. TDP-43 phosphorylation is characteristic of ALS/FTLD-TDP pathology and affects the phase separation, localisation, and degradation of TDP-43 in a site- and context-dependent manner [46]. A single phosphomimic at S48 can disrupt TDP-43 polymerisation, LLPS, and RNA splicing [50]. N-glycosylation of tau alters its biophysical properties in a glycan-size-dependent manner: small glycans promote tau aggregation and LLPS, whereas larger glycans attenuate these activities. Moreover, high-mannose glycans at N410 enhance tau phosphorylation by GSK3β, supporting a crosstalk between glycosylation and phosphorylation in AD-related tau biology [68].

Acetylation and oxidative modifications represent key post-translational regulators of TDP-43 phase behaviour. Acetylation of TDP-43 lysine residues impairs RNA binding and promotes aggregation, recapitulating pathological features observed in ALS and FTLD-TDP [49]. Oxidative stress further modulates TDP-43 LLPS through methionine oxidation within its LCD. H₂O₂-induced oxidation disrupts droplet stability and promotes condensate dissolution, an effect that can be reversed by methionine sulfoxide reductases [51]. Notably, the ALS-associated M337V mutation can attenuate this redox sensitivity, altering phase behaviour and aggregation propensity [51, 52]. Mechanistically, methionine sulfoxidation perturbs local helical structure and reshapes intermolecular interactions, impacting phase separation, amyloid formation, and chaperone engagement [52]. These findings identify redox-sensitive modifications as dynamic regulators of condensate properties and suggest a mechanism by which mitochondrial dysfunction, oxidative stress, and impaired redox buffering may drive pathological phase transitions.

### Mitochondrial metabolism, NAD^+^/NADH balance, and glucose hypometabolism

Mitochondrial dysfunction is a convergent feature of AD, PD, ALS, and Huntington’s disease and affects bioenergetics, ATP production, calcium buffering, balance of reactive oxygen species, and apoptotic signalling [93]. These processes can influence LLPS indirectly by changing ATP availability, redox turnover, protein oxidation, chaperone capacity, and local ionic homeostasis. The NAD^+^/NADH balance is particularly relevant because it coordinates mitochondrial respiration, redox buffering, sirtuin activity, PARP signalling, and stress responses. Reduced NAD^+^ levels are associated with ageing and neurodegenerative vulnerability. NAD^+^ boosting approaches are being explored as strategies to improve mitochondrial quality and cellular resilience to metabolic and proteostatic stress [94]. In the context of LLPS, this suggests a plausible but still incompletely validated model in which altered NAD^+^/NADH balance may influence phase behaviour indirectly through bioenergetic and redox changes, as well as through NAD^+^-dependent signalling pathways such as sirtuin-mediated acetylation control and PAR-dependent RBP condensation. Similarly, reduced cerebral glucose metabolism is a prominent feature of AD and may lower the neuronal energy supply, disrupt lactate metabolism and TCA-cycle activity, and increase oxidative stress. These metabolic disturbances could predispose neurons to pathological condensation, but direct evidence in human AD remains limited.

### Lipid metabolism and membrane-associated phase behaviour

Lipid metabolism is a critical but often underrepresented component of the metabolism–LLPS interface. Many neurodegeneration-associated proteins interact with membranes, lipid droplets, synaptic vesicles, or mitochondria-associated ER membranes. These interfaces can regulate the local concentration, conformation, nucleation, and aggregate growth of pathology-associated proteins. For example, α-synuclein binds synaptic vesicle membranes under physiological conditions, but lipid vesicles can promote α-synuclein aggregation through protein-lipid co-aggregation and fibril elongation mechanisms [74]. In synucleinopathy, lipid dyshomeostasis is increasingly recognised as a disease-relevant phenotype. Analyses on postmortem human samples and iPSC-derived neurons indicated changes of levels of specific phosphatidylserine species in brain regions most affected in PD and suggest a role for α-synuclein in lipid metabolism regulation at mitochondria-associated ER membranes [75]. These findings expand the metabolic perspective beyond soluble metabolites to include membrane composition and lipid microenvironments. Therefore, lipid remodelling may indirectly influence LLPS by changing membrane binding, interfacial protein crowding, vesicle clustering, and the likelihood of liquid-to-solid maturation.

## Condensate dysregulation and cognitive impairment

Protein aggregation and aberrant condensate maturation can impair neuronal function and are associated with cognitive decline in several neurodegenerative diseases [95]. However, an association with cognitive impairment should not be taken as a proof that LLPS is a causal mechanism. At the strongest end, causal inference is supported when a condensate-forming factor is genetically or experimentally manipulated, followed by behavioural or synaptic readouts. For example, RBM33 is elevated in aged hippocampal microglia, undergoes LLPS, binds the CDKN1A promoter, and promotes p21cip1-associated microglial senescence. Functionally, RBM33 depletion attenuates age-associated cognitive decline, whereas RBM33 overexpression exacerbates it, supporting a mechanistic RBM33 LLPS/p21cip1 axis in hippocampal aging [81].

Accumulating clinicopathological evidence links the pathological accumulation of phase-separating proteins to neurodegeneration. In patients with AD, concomitant TDP-43 pathology is associated with greater cognitive impairment, particularly when it involves limbic regions or co-occurs with hippocampal sclerosis [40, 96]. Interactions of tau and α-synuclein can also exacerbate cognitive decline. In vitro heterotypic LLPS studies show that the tau/α-synuclein droplets accelerate amyloid aggregation, offering a biophysical mechanism for overlapping neuropathologies in mixed dementias [97]. Nevertheless, available human data provide a direct link between aggregate burden and regional vulnerability to cognitive decline. Whether LLPS itself presents the proximal causal event in patients remains to be determined.

Although fragile X syndrome (FXS), caused by loss of fragile X messenger ribonucleoprotein (FMRP) function, is not a classical adult-onset neurodegenerative disorder, it provides a useful neurodevelopmental example of how neuronal condensates can influence cognition through local translation and mitochondrial dynamics. FMRP forms ribosome-rich condensates in axons and dendrites, is enriched at mitochondrial midzones, binds mitochondrial fission factor (MFF) mRNA, and supports local translation required for mitochondrial fission. Disruption of the FMRP–MFF axis results in synaptic dysfunction and cognitive deficits, which can be partially rescued by MFF overexpression [98]. Thus, FMRP is best viewed as a mechanistic example linking neuronal LLPS to cognitive phenotypes, rather than as direct evidence for classical neurodegeneration. In addition, cellular stress responses can shift from adaptive proteostasis to maladaptive condensate when they are prolonged or dysregulated. For example, ER stress-induced GRP78 condensates may help organise misfolded proteins during proteostatic stress, but sustained activation may promote aberrant protein metabolism and impair neuronal function [82]. Together, these examples illustrate how dysregulated condensate formation can influence neuronal function and highlight the need to determine how condensate composition and material properties shape disease-associated cellular and cognitive phenotypes in relevant models [9].

## LLPS and RNA metabolism/splicing regulation

RNA-binding proteins (RBPs) are central players in LLPS, forming ribonucleoprotein (RNP) condensates that regulate several aspects of RNA metabolism, including transcriptional coupling, splicing, RNA export, transport, translation, degradation, and stress-induced storage [7]. Dysregulation of these RNP condensates has been closely linked to the pathogenesis of neurodegenerative diseases because neurons are highly dependent on long-distance RNA transport, local translation, and activity-dependent remodelling of synaptic proteomes. Nuclear membraneless organelles, including nuclear speckles, paraspeckles, transcriptional condensates, and splicing condensates, can concentrate RBPs and RNA substrates to regulate alternative splicing. In the cytoplasm, stress granules and transport granules control mRNA triage, translational repression, and local protein synthesis. These condensates are likely associated with metabolism, because ATP depletion, oxidative stress, altered PAR signalling, and changes in RNA concentration can all reprogram RNP condensate dynamics and maturation [99].

The effect of aberrant LLPS on RNA processing is particularly prominent in TDP-43-related diseases. LLPS of TDP-43 contributes to the subcellular organisation and RNA-dependent functions of TDP-43. Mutations of TDP-43 or cellular conditions that impair RNA binding can alter both the condensation behaviour and RNA processing [53]. However, LLPS is not required for every aspect of TDP-43-mediated splicing, as phase-separation-deficient TDP-43 can retain splicing activity in selected assays. This suggests that LLPS fine-tunes, rather than universally enables, TDP-43-dependent RNA processing [100]. Nevertheless, specific structural elements can couple condensation to RNA processing. For example, conformational transitions within an α-helical region of TDP-43 during dimerisation are required for both phase separation and RNA splicing, and point mutations in this region disrupt both processes [101]. Furthermore, aberrant FUS condensation can also perturb RNA metabolism by sequestering RNAs and RNA-binding proteins, altering mRNA stability, and promoting persistent cytoplasmic assemblies in ALS/FTD contexts [102]. These results suggest that LLPS tunes RNA metabolism and spatial RNA organisation, whereas loss of normal condensate dynamics can produce RNA-processing defects.

## Therapeutic strategies targeting LLPS in neurodegeneration

**Fig. 4:**
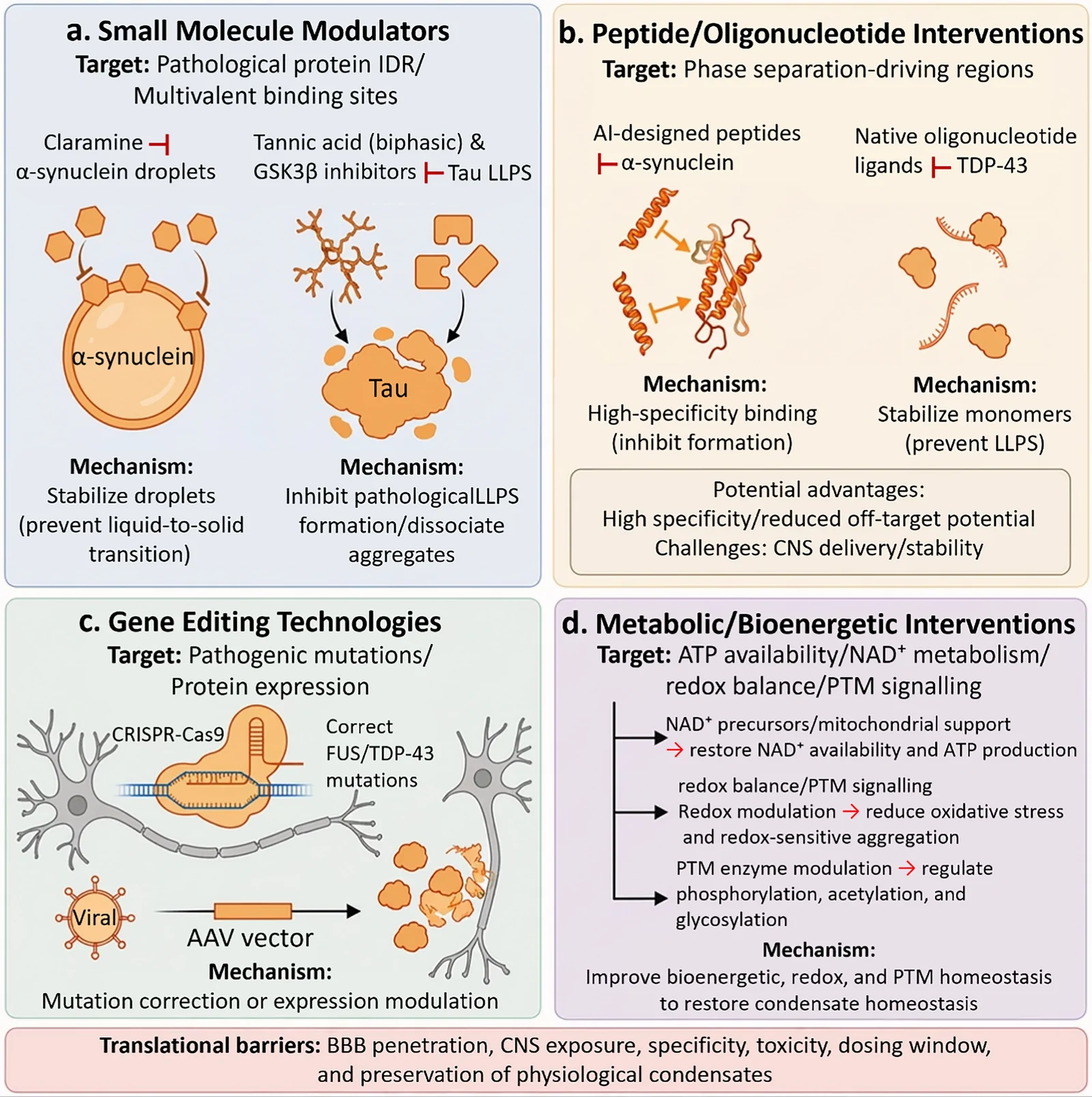
Emerging therapeutic strategies targeting LLPS in neurodegeneration. This schematic summarises emerging strategies to modulate aberrant LLPS and pathological protein aggregation. **a** Small molecules may modulate intrinsically disordered regions (IDRs) or multivalent interactions to stabilise condensates, prevent liquid-to-solid transition, or promote aggregate dissolution. **b** Peptides and oligonucleotides can disrupt the phase separation-driving interactions or stabilise monomeric/native states with higher molecular specificity. **c** Gene-editing, siRNA, and ASO approaches may correct pathogenic mutations or regulate the expression of aggregation-prone proteins and proteostasis factors. **d** Metabolic and bioenergetic interventions, including NAD⁺-boosting strategies, mitochondrial support, redox modulation, and PTM enzyme targeting, may indirectly restore condensate homeostasis by improving ATP availability, redox balance, and post-translational signaling. The translational feasibility of these approaches depends on BBB penetration, CNS exposure, specificity, toxicity, dosing window, and preservation of physiological condensates

Because phase separation contributes to the assembly and maturation of multiple aggregation-prone proteins in neurodegeneration, modulating the phase behaviour may provide therapeutic opportunities [1, 85, 103]. However, therapeutic development targeting phase separation is still at an early stage. Phase separation of macromolecules is a dynamic process and highly context-dependent. Most LLPS is driven by dysfunctional sequences that do not have a clear binding pocket for drug molecules. In the context of neurodegeneration, candidate molecules need to be delivered to CNS compartments or cross or bypass the BBB and, if feasible, selectively target pathological rather than physiological condensates. In this section, we summarise current LLPS-targeting research, distinguish mechanistic proof-of-concept from clinically mature interventions and highlight key translational barriers (Fig. 4).

### Small-molecule inhibitors and modulators of LLPS

Small molecules can, in principle, interfere with pathological phase separation by either preventing the liquid-to-solid transition along the pathogenic pathway or promoting dissolution of pathological condensates [104, 105]. For example, the small molecule claramine can stabilise α-synuclein condensates and inhibit its aggregation both in vitro and in PD models [106]. Additionally, phenolic compounds, such as forsythoside B, echinacoside, and 4-hydroxyindole, can inhibit α-synuclein aggregation by increasing the condensate fluidity, reducing the fibril-induced toxicity, disaggregating preformed fibrils, and attenuating α-synuclein proteotoxicity. Forsythoside B and echinacoside can improve locomotor deficits and lifespan in a *C. elegans* model of PD [107]. However, their clinical translation requires further validation of BBB penetration, pharmacokinetics, brain target engagement, dose–response relationships, and long-term safety.

Tannic acid has been identified as a biphasic modulator of tau LLPS. At low tannic acid-to-tau ratios, it can promote tau phase separation, whereas at higher ratios it suppresses LLPS, likely by changing the hydrophobic interactions and hydrogen-bonding networks [70]. GSK3β-mediated phosphorylation of the tau proline-rich domain is critical for tau condensation [67]. Together, these findings indicate that LLPS intervention can be achieved through complementary approaches, including direct condensate-modifying compounds that alter condensate properties and enzyme-targeting strategies that regulate condensate formation through PTMs. For tau and α-synuclein, such condensate-modifying strategies are conceptually attractive, but clinical translation will require further pharmacological validation, disease-stage-specific dosing, verification of brain target engagement, and reliable biomarkers of condensate regulation in the human brain [64, 103–107].

For TDP-43, stabilising the native-state conformations represents a promising strategy to limit pathological aggregation [108]. C-terminal substitutions, such as S333D/S342D, can maintain the monomeric state and prevent fibrillation and aberrant phase separation of TDP-43 without altering its physiological function. Native oligonucleotide ligands have been shown to stabilise TDP-43, indicating the potential of nucleic acid-based interventions. Halogen-doped graphene quantum dots can suppress TDP-43 phase separation and amyloid formation under oxidative stress via hydrophobic and electrostatic interactions [109]. However, the clinical translation of nanomaterial-based TDP-43 modulators requires careful evaluation of their CNS delivery, biodistribution, clearance, long-term toxicity, immune activation, reproducibility, and scalability. More broadly, TDP-43-targeting interventions are expected to suppress pathological condensation while preserving its physiological roles in RNA metabolism and stress responses.

### Other emerging therapeutics

Other strategies indirectly targeting LLPS-related pathways have been investigated (Table 1). These include targeting PTM enzymes, enhancing proteostasis pathways, modulating microenvironmental properties within condensates (e.g., viscosity, polarity, pH), and restoring cellular energy metabolism to reduce the probability of pathological liquid-to-solid transitions. A particularly relevant metabolic strategy is ATP supplementation through mitochondrial and NAD^+^/NADH pathways. In neurons, intracellular ATP depletion promotes axoplasmic viscosity and pathological protein condensation, and increasing the NAD^+^/NADH-related metabolic capacity may help maintain ATP production and protein homeostasis [17, 94]. Nonetheless, NAD^+^ precursors or metabolic boosters should be viewed as upstream modulators rather than direct LLPS-targeting drugs, unless target engagement and condensate-level effects are demonstrated in disease-relevant CNS models.

**Table 1:** Therapeutic agents targeting pathological LLPS

Strategy type	Mechanism	Targets	Advantages	Challenges
Small-molecule modulators	Prevent pathological LLPS, stabilize liquid state, or promote dissolution of aggregates; representative evidence includes claramine, phenolic compounds, tannic acid, GSK3β inhibition, and graphene quantum dots [67, 70, 106, 107, 109]	Claramine (α-synuclein), phenolic compounds (α-synuclein), tannic acid (tau), GSK3β inhibitors (tau), graphene quantum dots (TDP-43)	Cell permeability; potential for high-throughput screening; can target “undruggable” proteins	Specificity; BBB penetration; brain exposure; off-target effects; toxicity; identifying optimal dose for biphasic modulators
Targeted molecular interventions (Peptides/Oligos)	Specific binding to proteins or nucleic acids to interfere with LLPS-driving interactions or stabilize native states [108]	AI-designed peptides (α-synuclein), Native oligonucleotide ligands (TDP-43)	High specificity; reduced off-target effects; can be rationally designed	Delivery to CNS; stability in vivo; immunogenicity; endosomal escape; avoiding interference with physiological RNA metabolism
Gene editing	Corrects pathogenic mutations, modulates protein expression, introduces protective modifications, or enhances chaperone/proteostasis pathways [1, 95]	Correcting FUS/TDP-43 mutations; downregulating α-synuclein/tau expression; introducing phosphomimics; enhancing HSP70	Addresses root genetic causes; potential for long-lasting effects	Targeted delivery to specific neuronal populations; off-target edits; immunogenicity; reversibility; ethical considerations; current stage remains nascent
Metabolic/proteostasis modulation	Supports ATP production, NAD^+^/NADH balance, redox buffering, chaperone capacity, or PTM homeostasis to reduce pathological liquid-to-solid transition risk [17, 94]	NAD^+^ precursors, mitochondrial support, kinase/PTM enzyme modulators, chaperone-enhancing approaches	Targets upstream vulnerability; may improve broader neuronal resilience; compatible with combination therapy	Indirect mechanism; uncertain condensate-level target engagement; dosing and disease-stage dependence; systemic metabolic effects; needs CNS biomarkers

The increasing understanding of LLPS is reshaping therapeutic thinking in neurodegenerative diseases. Small-molecule modulators, peptides, oligonucleotides, nanomaterials, gene-editing tools, and metabolic interventions can alter the phase behaviour or the upstream modulators in experimental systems. The major translational barriers include BBB penetration, CNS exposure, cell-type specificity, off-target effects, toxicity, compound stability, chronic dosing, and the lack of validated biomarkers for condensate modulation in patients. Therefore, future studies on LLPS-targeting therapy should combine biophysical assays, pharmacokinetic/pharmacodynamic analyses, and human biomarker development.

## Conclusion

Studies of LLPS have reshaped our understanding of intracellular organisation and its disruption in neurodegenerative diseases. While previous work primarily focused on the protein-intrinsic properties that drive condensate formation, emerging evidence highlights the importance of the cellular context, particularly metabolic regulation, in modulating phase behaviour. Metabolic regulation, including bioenergetic status, ATP availability, NAD^+^/NADH balance, redox state, lipid composition, enzyme activity, and stress responses, plays a critical role in condensate formation, dynamics, and pathological maturation. Pathological phase separation can promote protein aggregation and may disrupt RNA metabolism, synaptic homeostasis, proteostasis, and cognitive function, thereby exacerbating neurodegenerative processes. Yet metabolic dysregulation should be considered as a context-dependent regulator rather than a well-established upstream cause of pathological LLPS. Future research should move beyond observational studies to establish causal links between defined metabolic dysfunction, condensate material properties, and disease-relevant phenotypes across neurons, glia, animal models, and human tissues. From a translational perspective, metabolism-guided LLPS interventions will need to achieve CNS exposure while preserving physiological condensate functions, which need integrated biochemical, biophysical, metabolic, pharmacological, and in vivo validation.

## Data Availability

Not applicable.
